# Supplementary material for: Patterns of Intron Gain and Loss in Fungi
Source: PLoS Biol. 2004 Nov 30;2(12):e422. doi: 10.1371/journal.pbio.0020422 (PMC532390; doi:10.1371/journal.pbio.0020422)
Supplement: Table S1 — Also available at http://genes.mit.edu/NielsenEtAl/. (4.3 MB ZIP). [file pbio.0020422.st001.zip › NielsenEtAl/html/874.html]

AN6911.1.NCU06268.1.MG05256.1.FG02565.1


```
 CLUSTAL W (1.82) Multiple Sequence Alignments - Introns Inserted


Sequence 1: NCU06268.1	870 aa
Sequence 2: MG05256.1	829 aa
Sequence 3: FG02565.1	793 aa
Sequence 4: AN6911.1	774 aa
Alignment Length: 876 aa
Number Identitical Residues: 336 aa
Alignment Score (without introns) 19427


MG05256.1 	MELS-PTAGWEQVGDKYYRKVQLYTEVFDLDLDLDNYVVAGAPDGGAVA~LYL------D
NCU06268.1	MEVPHPTAGWEQVGEKFYRKTQLYTQVFDADLDLDNYIVAGAPYGGAVG~IVFLVIPLDD
FG02565.1 	------------------------MSSNSHQTELTNLLALWR-------~---------D
AN6911.1  	MAPSNPLANWERLGSSFYRKVPLYHSVFGEDVELENYIVAGAPYGGAIA1LYR------D
          	   . . :. .  ...   .     .  . : :* * :.   . ..: .          *

MG05256.1 	ENKLVE~YRAGKARKPSIDIYSCAGKLIRSIN~WDKGSIRGLGWSEDEKLLVVTKDGTVR
NCU06268.1	DVFQKA1FAAGRPSKPSIDIHSCAGKLIRRIP~WDKGSIKGLGWSEDEKLLVVTGDGTVR
FG02565.1 	DTKLLA~YQPGRSAKPAIDIYSLAGKKLRSIP~WDNGTIKGLGWSEDETLLVVTADGNVR
AN6911.1  	ESKPLR~FRDAQTGKSNIDIYSRSGKLINRLN0WEHGTIRGLGWSDQEELLVITEDGTVR
          	:      :  .:. *. ***:* :** :. :  *::*:*:*****::* ***:* **.**

MG05256.1 	CYYGLQEDFTQFSLGH~GAEEYGVHSCR~FYETGMVALLTNNTLVTVSSYEEPRPKPLVS
NCU06268.1	CYYDLQGDFTQFSLGH~DADEIGVRSCK~FYGHGLVALLNNNSLVSVSAYDEPRPKLLAQ
FG02565.1 	CY-DLQGEFTQFSLGH~GADNYSVESCR2FYDHGMVALLGNNTLITVSSYTEPRPKALAQ
AN6911.1  	RYFGLYGDFTSFSLGN0GAEEYGVRACR~FWNSGFVALLANNQLIAVSSYNEPRPRLLAQ
          	 * .*  :**.****: .*:: .*.:*: *:  *:**** ** *::**:* ****: *..

MG05256.1 	PPEGEVHSWTIIAPRYTLSRSVEVLLSIGQTIYVTDASECEDRYLDIGPFTHMAVSPNGK
NCU06268.1	PPEGPVHSWSLVPPAYTLSRSVEVLLSIGQTIYVSDASECEDRFIDIGPFSHISISPNGK
FG02565.1 	TPEGGINAWSIISPNHTLSRSVEVLLGVGKTVYVVDATDCEDRFLDIGPFSHISVSPDGR
AN6911.1  	CPEGEVASWSLIPPAYTLSRSVEVLLAVDKTIYLVDPTEAEDKVLQNGPFKHASVSPTGQ
          	 *** : :*:::.* :**********.:.:*:*: *.::.**: :: ***.* ::** *:

MG05256.1 	FAALYTPAGEVHVITCDFQTRLSEHDLKSKIPPKYLRWCGNDAVVIAWEDEIHLVGPGAA
NCU06268.1	FVALYTKTGKAHVISSDFQTRLSEYVSKSKIPPQYFEWCGNDAVVIAWEDEVRLVGPGGS
FG02565.1 	YVNLYAANGKAHVISSDFQERLFEHDSNSQTPPLYVEWCGSDA-LIAWEDEVHIIGPGDS
AN6911.1  	FVALLTAEGKVWVVSSDFQSKYSEYDPDSRVTPRAVEWCGNDAVVIAWEDEVHLIGPNGA
          	:. * :  *:. *::.*** :  *:  .*: .*  ..***.** :******::::**. :

MG05256.1 	VAT~FFY-NGRIHIVQD~FDGVRVLTNETCDFLQKVPD~VTEEVFRIGTES-AASILLDA
NCU06268.1	LAR~FYYDNGRIHILPD~FDGVRVLANDTCDFLQKVPD~VIEEVFCLGTDS-PASILLDA
FG02565.1 	SSS~YIYDSTRVHVVSE1HDGARLITNDFCEFLERVPR~DALEVFGQSSDSSPASILLDA
AN6911.1  	AAR2YWY-DGTVHVLPE~FDGVGLLTNDTYEFLHKVSD1VTETIFRLGSTS-PASVLLDS
          	 :  : * .  :*:: : .**. :::*:  :**.:*.      :*  .: * .**:***:

MG05256.1 	VEQLEMQSPKADDNIQLIRANLTEAVDTCVNAAGQEFNVHWQKQLLKAASFGKSVLDIYN
NCU06268.1	VEQLEMQSPKADDNIQLIRSNLVEAVDTCVNAAGQEFSIHWQKQLLKAASFGKSVLDIYN
FG02565.1 	VGQLEVQSPKADDYIQLIRPNLTGAVDTCVNAAGREYDTHWQKRLLKAASFGKSVLDIYN
AN6911.1  	IDLLEKKSPKADDNIQRIKPSLPEAVDMCVKASGLEFDAYWQKRLLKAASFGKSVLDLYN
          	:  ** :****** ** *:..*  *** **:*:* *:. :***:*************:**

MG05256.1 	SDDFVDMCETLRVLNAVRFYEVGLSLSFDQYQRLGPDRLIGRLLDRHDYLLALRIAGYLR
NCU06268.1	SDDFVDMCETLRVLNAVRFFEIGLPLSYEQYQRLSPSGLIARLLNRREYLLALKIAGYLR
FG02565.1 	SDEFVDMCETLRVLNAVRYYEVGMPLSFEQYHRLTPEALIRRLLNRHEYLLALKIAGYLK
AN6911.1  	SDEFVEMTEKLRVLQAVRDFQIGLAVSYDQYMRLTSEKLIERLVNRRNYLLAIRISEYLQ
          	**:**:* *.****:*** :::*:.:*::** ** .. ** **::*::****::*: **:

MG05256.1 	LPTDRIYVHWACAKVRVSSDDDDTIARVVVDKLSGKPGISFEAIARAAYDEGRTRLATEL
NCU06268.1	LPTDKIYVHWASAKVRVGAEDDDTICRKIVDKLSGKPGISFEAIARAAYDEGRGRLATEL
FG02565.1 	LPTDRIYVHWASSKVRVGGEDDDTICRLIVERLSGKPGISFEEIARSAYQEGKGRLATEL
AN6911.1  	LPADKIYVHWGSSKVKVSTVDDEAVCKLIVQRLDGKPGISFEAIAQAAYDEGRSHLATQL
          	**:*:*****..:**:*.  **:::.: :*::*.******** **::**:**: :***:*

MG05256.1 	LNHEPRAGRQVPLLLSMDEGEVALDKAVESGDTDLVLSVLHELRRKLPLASFFRTINRRP
NCU06268.1	LNHEPRAGRQVPLLLSMEEDEVALDKAIESGDTDLVYFVLHQLKRKLPLATFFRVISSRP
FG02565.1 	LNHEPRGGKQVPLLLSMEEDELALDKAVESGDTDLILSVLLQLRKKLPLAAFFRVINARP
AN6911.1  	LNHEPRAGKQVPLLLNMEEDEIALDKALQSGDDDLVNYVLLHLKSKLPIASFFRMINTRP
          	******.*:******.*:*.*:*****::*** **:  ** .*: ***:*:*** *. **

MG05256.1 	VATALVEAAA----------VREG--------------------DNALLKDLYYQDDRRL
NCU06268.1	TATALVEASARLGGGVVLGNVDEGHHAASHSYSQSGSGSTTTCEDTALLKDLYYQDDRRV
FG02565.1 	AATALVEALA----------MEEG--------------------DNTLLKDLYYQDDRRT
AN6911.1  	MASALVETTAREE-------------------------------DPELLKDLFYQDDRPL
          	 *:****: *                                  *  *****:*****  

MG05256.1 	DGASVFLREAIQQPDARTAGDKMALAARLLSDNARESAFELR--AIKEAATLLRMQEAFD
NCU06268.1	DGSDVFVREALRQPEARTAADKLALAAKLLSDNAKENAFELS--ALKEATTLLRMQEAFD
FG02565.1 	DGANIFIRESLKQPDARTSSDKLTLAAKLLAD-SKESSFEVH--ALKEATTLLRMQEAFD
AN6911.1  	DGSNVLLSDALKESDLPRKQEKLLLASRLLSD-SKDPTLVLHRNLLNESSQLLKTQEALD
          	**:.::: :::::.:     :*: **::**:* ::: :: :  . ::*:: **: ***:*

MG05256.1 	RDLT--DSFTGLSVNETMFKLIRLGYHGRAKKIQSEFKVPERVACWIR2LRALVAKRDWN
NCU06268.1	RDLHP-ETFTGLSVNQTVFKLIRLGYHGRAKKVQSEFKVTEKVAWWIR2LQALVSKRDWS
FG02565.1 	RDLT--DTFTGLSVNETMFKLIRLGYHGRAKKIQSEFKVPEKVAWWIR2LRALVAKRDWN
AN6911.1  	KDLAERSEFLGLSLNETVYRLIRSGYGRRAQKIQSDFKMPERTYWWLR~LRALVAKRDWG
          	:**   . * ***:*:*:::*** **  **:*:**:**:.*:.  *:* *:***:****.

MG05256.1 	EIEEIS-KARKSPIGWE0PYFNLVLQAGNPRLASVFIPKCTN---LKDVTTIVMYEKCGM
NCU06268.1	EIEDLASKNRKSPIGWE~PFYTQVLQAGNQRLAATFIPKCAASGGLEPGATIGMYEKCGM
FG02565.1 	EIEEIS-KTRKSPIGWE0PFFNLTLQAGNPRLASVFVPKCTA---IEAGETITMYEKCGM
AN6911.1  	ELEEIG-KIKKSPIGWE0ELETQSWLPSSSQNAPIYPRRKEL--------------RCG-
          	*:*::. * :*******    .    ... : *. :  :                 :** 

MG05256.1 	RIKAAEEAVRLKNRDEWARLIEAAGKNTTEGREIERVGAAAFKRGV
NCU06268.1	RVKAAEEAVKFKDAEAWARLLEEAGRGTQEGREIERLGQSVFRR--
FG02565.1 	RVKAAQEAVRLKDSESWARLLEDAGKGSQEGREIERLGQTAFKK--
AN6911.1  	----------------------------------------------
          	
```
